# Particulate matter-induced hypomethylation of *Alu* and *LINE1* in normal human bronchial epithelial cells and epidermal keratinocytes

**DOI:** 10.1186/s41021-022-00235-4

**Published:** 2022-02-16

**Authors:** Ji Yun Lee, Won Kee Lee, Dong Sun Kim

**Affiliations:** 1 Department of Anatomy and BK21 Plus KNU Biomedical Convergence Program, Daegu, Republic of Korea; 2 Preventive Medicine, School of Medicine, Kyungpook National University, Daegu, Republic of Korea; 3 Department of Anatomy, School of Medicine, Kyungpook National University, 2-101 Dongin-dong, Jung-gu, 702-422 Daegu, Republic of Korea

**Keywords:** Particulate matter, DNA methylation, WGBS, Alu, LINE1, Subfamily

## Abstract

**Background:**

Airborne particulate matter (PM), a complex mixture of organic and inorganic compounds, is a major public health concern due to its adverse health effects. Understanding the biological action of PM is of particular importance in the improvement of public health. Differential methylation of repetitive elements (RE) by PM might have severe consequences for the structural integrity of the genome and on transcriptional activity, thereby affecting human health. This study aimed to evaluate the effect of inhaled and non-inhaled PM (PM_2.5_, PM_10_, and PM_10_-PAH) exposure on DNA methylation. We quantitatively measured the methylation content of *Alu* and *LINE1* in PM-treated normal human bronchial epithelial cells (NHBE) and normal human epidermal keratinocytes (NHEK) by using whole-genome bisulfite sequencing and pyrosequencing.

**Results:**

All PMs exposure significantly lowered *Alu* and *LINE1* methylation in both cells than in mock-treated controls. Hypomethylation was more prominent in PM_10_-PAH exposed-NHBE and PM_10_ exposed-NHEK. *Alu* and *LINE1* methylation change exhibited different sensitivity according to the subfamily evolutionary ages, with stronger effects on the oldest L1-M and Alu J in NHBE, and oldest L1-M and youngest Alu S in NHEK.

**Conclusions:**

These results demonstrate that the differential susceptibility of PM-induced hypomethylation of *Alu* and *LINE1* depends upon RE evolutionary age and PM type.

**Supplementary Information:**

The online version contains supplementary material available at 10.1186/s41021-022-00235-4.

## Introduction

Air pollution has become an important health concern and ranks as the sixth leading risk factors for premature death globally [1, 2]. Exposure to air pollution is ubiquitous and typically beyond the control of individuals, and the resulting health burden on the population can be high. Particulate matter (PM), one of the most toxic forms of air pollution, is recognized as a major health hazard worldwide, and is associated with respiratory, cardiovascular, and skin diseases [3–5]. However, the mechanisms linking PM exposure to adverse health outcomes have not been completely clarified. The size and composition determine the toxicity of the particle [6]. PM consists of a mixture of volatile organic compounds, polycyclic aromatic hydrocarbons (PAH) and inorganic chemicals such as heavy metals that, both individually and together, cause adverse health effects. PM constitutes of microscopic particles of solid or liquid matter suspended in the air. Airborne PM is usually classified as coarse PM_10_ (<10 mm) and fine PM_2.5_ (<2.5 mm), depending on the aerodynamic diameter of the particles. PM_10_ is composed of inhalable particles from dusts, industrial emissions, and traffic emissions. PM_2.5_ is primarily composed of organic carbon compounds, nitrates, and sulfates. Understanding the biological action of PM is of particular importance in improvement of public health.

Alterations in DNA methylation are associated with various health outcomes, representing an interface between the environment and human disease [7]. Emerging data indicate that PM exposure modulates DNA methylation, a major genomic mechanism of gene expression control, and that these changes might in turn influence inflammation, disease development, and exacerbation risk [8]. However, whether such effects are targeted to specific sites or scattered across the genome globally remain challenging. *Alu* and *long interspersed nucleotide element 1* (*LINE1, L1*) are significant components of repetitive transposable DNA elements, constituting approximately 17% and 11% of the human genome, respectively [9], representing as a surrogate marker for genome-wide global methylation levels. Interestingly, transposable repeats are considered as a responser to environmental stressors [10, 11] and their reactivation through hypomethylation can increase genome instability, reactivate lowly expressed genes, or disrupted gene function, thereby potentially contributing to disease-related pathological consequences [12, 13] and provide promising candidate biomarkers for human disease including cancer [14]. Unfortunately, PM-induced DNA methylation of repetitive elements (RE) reported in most previous studies is measured in blood cells, representing overall results from the body organs [15–18]. However, methylation in skin and lung, major targets of air pollution, has not been well studied. In the present study, we treated the normal human bronchial epithelial cells (NHBE) and normal human epidermal keratinocytes (NHEK) with inhaled and non-inhaled PM (PM_2.5_, PM_10_, and PM_10_-PAH) and then determined the changes in global DNA methylation using whole-genome bisulfite sequencing (WGBS) and pyrosequencing.

## Materials and methods

### PM preparation

PM_2.5_, which is a standard diesel PM (SRM1650b) issued by the National Institute of Standard and Technology (Gaithersburg, MD, USA), was bought from Sigma-Aldrich (St. Louis, MO, USA). It was dissolved in dimethyl sulfoxide (DMSO) at 50-mg/ml concentration. PM_10_-like fine dusts (ERM-CZ100 and ERM-CZ120), which are issued by the European Reference Materials (ERM, Belgium), were brought from Sigma-Aldrich. The former (PM_10_-PAH) includes several PAHs (benzoanthracene, benzopyrene, benzofluoranthene, and dibenzoanthracene, etc.) in ambient PM_10_, the latter (PM_10_) contains heavy metals (arsenic, cadmium, lead, and nickel). They were suspended in phosphate buffered saline (PBS) at 5-mg/ml concentration. PM was prepared just before cell application and sonicated in an ultrasonic bath for 10 min to avoid variability in PM composition and aggregation of particles.

### Cell culture and PM treatment

NHBE and NHEK were obtained from the American Type Culture Collection (ATCC, Manassas, VA, USA). NHEK (ATCC PCS-200-011) was grown in Dermal Cell Basal Media (ATCC PCS-200-030) supplemented with Keratinocyte Growth Kit (ATCC PCS-200-040) to propagate in serum-free conditions. NHBE (ATCC PCS-300-011) was cultured in serum-free Airway Epithelial Cell Basal Media (ATCC PCS-300-030) supplemented with Bronchial Epithelial Cell Growth Kit (ATCC PCS-300-040). Both cells were grown at 50% confluency and were treated with PM_2.5_, PM_10_, and PM_10_-PAH for 3 days at a final 50-µg/ml concentration of without a medium change. Cells maintained in culture medium with vehicle (0.1% DMSO or 1% PBS) were used as untreated control groups.

### WGBS library preparation and sequencing

The cells were washed with PBS, and genomic DNA was extracted using a QIAamp DNA Mini Kit (Qiagen, Valencia, CA, USA) according to the manufacturer’s instruction. The concentration and quality of the DNA were determined using an Agilent Bioanalyzer 2100 (Agilent Technologies, Santa Clara CA, USA) and agarose gel electrophoresis. DNAs were fragmented using a Bioruptor (Diagenode, Liege, Belgium) to an average size of approximately 250 bp, followed by the blunt ending, 3’-end addition of dA, and adaptor ligation (in this case of methylated adaptors to protect from bisulfite conversion). Ligated DNA was bisulfite-converted using the EZ DNA Methylation-Gold kit (Zymo Research Corp, Irvine, CA, USA). Fragments pf length 200–250 bp were excised from a 2% TAE agarose gel, purified using a QIAquick Gel Extraction Kit (Qiagen), and then amplified via PCR. Libraries were constructed from PCR products with BGI’s DNA nanoball (DNB) technology. The qualified libraries were sequenced using the DNBSEQ®-platform (BGI, Shenzhen, China). Base-calling was performed using the BGISEQ-500 software (v 0.3.8.1111).

### Data filtering

Data filtering was conducted using the elimination of contaminating DNA and low-quality reads. Low-quality reads include three types and the reading that accord with one of them will be removed: (1) contain adaptor sequence; (2) N-base number >10%; (3) the number of bases with a quality of <20% and >10% was trimmed. Only clean data was used for further analyses.

### Reads mapping and differentially methylated level analysis

Clean reads of each sample were mapped to human UCSC hg19 reference genome using BSMAP software (v2.90) to obtain BAM file. BAM files were sorted and indexed using Samtools software (v0.1.18). The parameters in the mapping and the results of mapping were shown in Supplementary Table 1. Methylation level was determined by dividing the number of reads covering each methylcytosine by the total reads covering the cytosine. MOABS software (v1.3.2) was used to calculate the methylation level of every cytosine in every sample, and to determine differentially methylated cytosine (DMC). After calculating the methylation level, cytosine was considered as “hypomethylated” when the methylation level of cytosine ≤0.2, and Fisher’s Exact Test *p*-value was <0.05.

### *Alu* and *LINE1* methylation analysis

To analyze the methylation level of six evolutionary subfamilies in Alu (Alu Y, Alu S, and Alu J) and LINE1 (L1-H, L1-P, and L1-M), genomic coordinates of all repeats based on hg19 were extracted and obtained from UCSC genome browser using RepeatMasker track as Sae-Lee et al. [19]. All subtypes of 6 target repeats (for example, AluJb, AluJo, AluJr, etc. for AluJ subtypes) were extracted, and methyl-cytosine data within 6 repeats were collected from DMC data of MOABS results in each sample using Python scripts. Data analysis workflow was shown in Supplementary Fig. 1. All CG sites in hg19 and DMCs within six repeats were counted through the samples.

### Pyrosequencing of *Alu* and *LINE1*

Bisulfite-converted DNA was amplified with PCR primers under previously described conditions [20]. The PCR products were then assayed on the PyroMark Q24 with PyroMark Gold Q24 Reagents (Qiagen) and then analyzed with accompanying software. The degree of methylation was expressed for each DNA locus as a percentage of methylated cytosines over the sum of methylated and unmethylated cytosines. We used non-CpG cytosine residues as built-in controls to verify the bisulfite conversion. In every pyrosequencing run, three controls were included. One well was filled with water to ensure no contamination, and two wells were filled with CpGenome universal methylated and unmethylated DNA (Chemicon, Temecula, CA, USA) as positive and negative control to weigh the repeatability of the assay.

### Statistical analysis

Data are presented as the means ± standard error (SE) of three independent experiments. One-way ANOVA was used for the mean difference test between the groups, and Bonferroni’s correction *p*-value was used for post-hoc comparison the two groups when the ANOVA was significant. The statistical analyses were conducted using SAS 9.4 (SAS Inc., Cary, NC, USA), and the plots were constructed using R version 4.1.0 (The R Foundation for Statistical Computing, Vienna, Austria). The significance level of the statistical test was set to 5%.

## Results

**Fig. 1:**
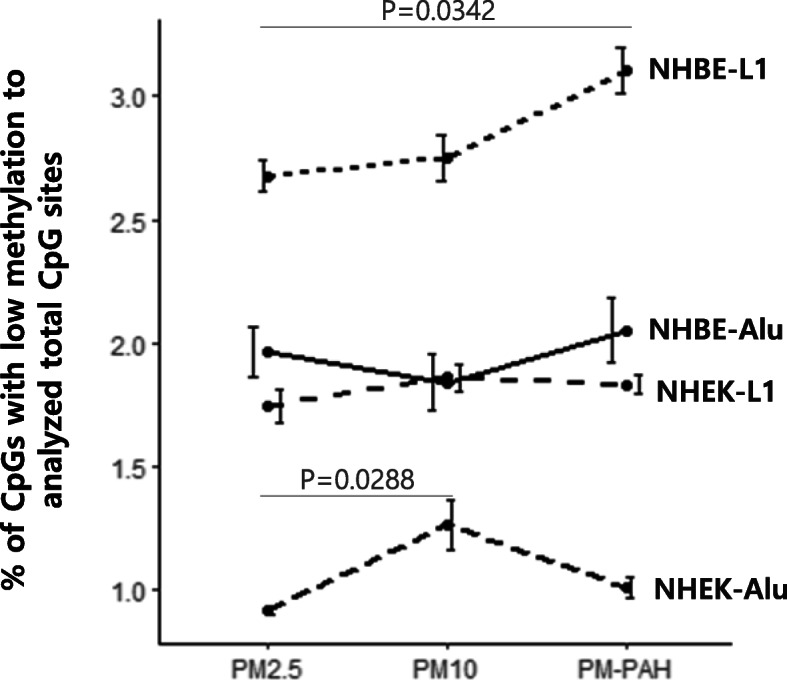
*Alu* and *LINE1* methylation change in NHBE and NHEK after PM exposure. The hypomethylation ratio was calculated as a percentage of CpGs with low methylation to analyzed total CpG sites and the means ± SE of three independent WGBS experiments are shown. The mean methylation ratios of PM-treated cells were compared to mock-treated cells using one-way ANOVA, and Bonferroni’s correction *p*-value was used for post-hoc comparison between two groups where the ANOVA was significant

**Fig. 2:**
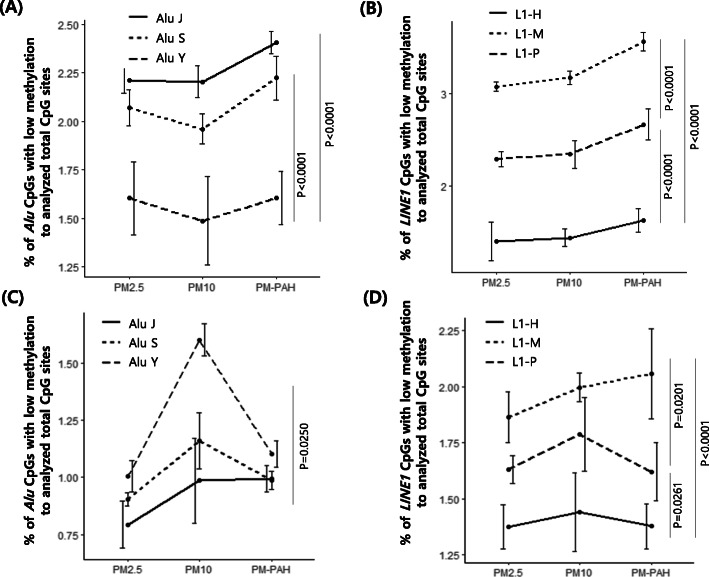
Differential hypomethylation of *Alu* and *LINE1* evolutionary subfamilies in NHBE and NHEK following PM treatment. (A) *Alu* change in NHBE. (B) *LINE1* change in NHBE. (C) *Alu* change in NHEK. (D) *LINE1* change in NHEK. The means ± SE of three independent WGBS experiments are shown. Bonferroni-adjusted *p*-value was the result of comparing between the two groups

**Fig. 3:**
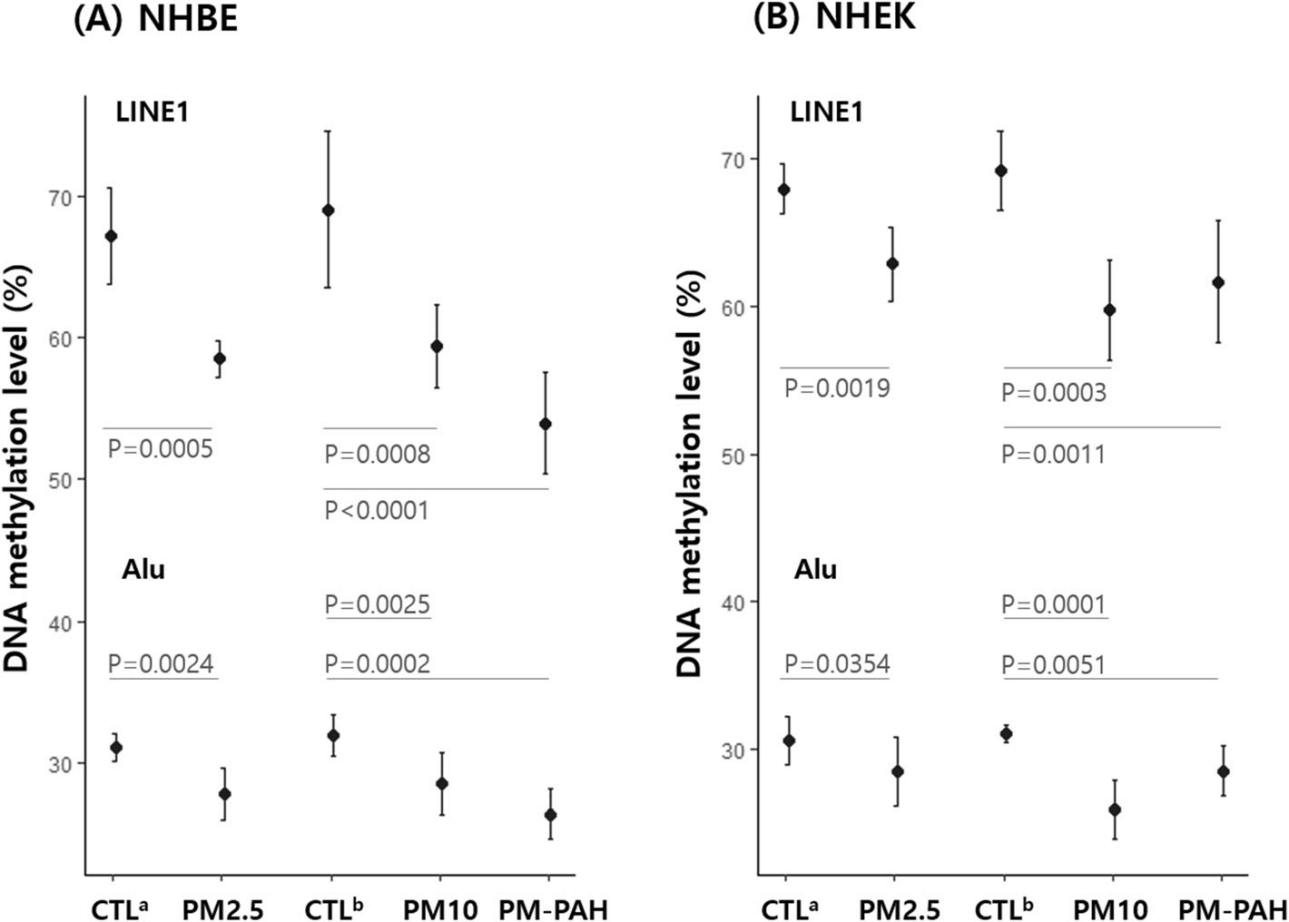
Distribution of *Alu* and *LINE1* methylation level in PM-exposed NHBE (**A**) and NHEK (**B**). Methylation level was expressed as a percentage of 5-methylcytosine divided by the sum of methylated and unmethylated cytosines. The mean ± 95% confidence interval of three independent pyrosequencing is shown. The comparisons of mean methylation levels were evaluated using ANOVA. Bonferroni-adjusted *p*-value was the result of comparing between the two groups. Cells maintained in culture medium with vehicle were used as control group (CTL^a^, 0.1% DMSO; CTL^b^, 1% PBS)

We comprehensively examined the effect of inhaled and non-inhaled PM exposure on RE *Alu* and *LINE1* by WGBS. Based on the reference genome and the UCSC RepeatMasker, approximately 34.6% of all 28 million CpG sites are in *Alu* (25.0%) and *LINE1* (9.6%). The RepeatMasker library mapped 7,040,695 *Alu* and 2,651,373 *LINE1* loci in the UCSC hg19 reference genome assembly, corresponding to 10.1% and 17.1% of the human genome respectively (Table 1). Because human *Alu* and *LINE1* are heavily methylated in normal tissues, all PMs exposure significantly increased the unmethylated CpGs of *Alu* and *LINE1* in both NHBE and NHEK comparing with mock-treated (0.1% DMSO or 1% PBS) cells (Table 1), indicating a potentially defective functionality of these RE. Moreover, PM-induced hypomethylation was prominent in NHBE compared with NHEK as well as in *LINE1* compared with *Alu* (Fig. 1). Although there was a narrow margin between each PM, PM_10_-PAH and PM_10_ exhibited the strongest effect on RE hypomethylation in NHBE and NHEK, respectively (Fig. 1). Furthermore, because CpG content and DNA methylation levels dramatically differ across subfamilies, we evaluated the sensitivity of DNA methylation in differentially-evolved *Alu* and *LINE1* subfamilies to different types of airborne PM. We subdivided *Alu* and *LINE1* into three evolutionary subfamilies; oldest Alu J and L1-M, intermediate Alu S and L1-P, and youngest Alu Y and L1-H. Interestingly, Alu J and L1-M showed the strongest hypomethylation in NHBE following treatment with three PMs, whereas Alu Y and L1-M exhibited the strongest hypomethylation in the NHEK (Fig. 2), indicating the association of differential susceptibility of the RE hypomethylation with evolutionary ages of subfamilies. In addition, although pyrosequencing is not expected to comprehensively reflect DNA methylation patterns within individual subfamilies, we have validated the WGBS results by pyrosequencing for accuracy and reproducibility of methylation levels. Significantly reduced methylation of *Alu* and *LINE1* was detectable in both NHBE and NHEK following all PMs exposure (Fig. 3). Likewise, PM_10_-PAH and PM_10_ exhibited the strongest effect on RE hypomethylation in NHBE and NHEK, respectively (Fig. 3), showing that a similar trend might be present between WGBS and pyrosequencing methods.

**Table 1:** PM-induced hypomethylation of *Alu* and *LINE1* in NHBE and NHEK

	L1-H			L1-P			L1-M			L1		
	**PM**_**2.5**_	**PM**_**10**_	**PM**_**10**_ **-PAH**	**PM**_**2.5**_	**PM**_**10**_	**PM**_**10**_ **-PAH**	**PM**_**2.5**_	**PM**_**10**_	**PM**_**10**_ **-PAH**	**PM**_**2.5**_	**PM**_**10**_	**PM**_**10**_ **-PAH**
**Unmethylated CpGs** **in NHBE**	604	622	702	29,409	30,050	34,696	40,765	42,033	49,037	70,778	72,706	84,603
**Unmethylated CpGs** **in NHEK**	591	619	592	20,902	22,228	20,774	24,707	26,474	27,270	46,201	49,322	48,637
**Analyzed total CpGs**	43,045			1,282,366			1,325,962			2,651,373		
	**Alu Y**			**Alu S**			**Alu J**			**Alu**		
	**PM**_**2.5**_	**PM**_**10**_	**PM**_**10**_ **-PAH**	**PM**_**2.5**_	**PM**_**10**_	**PM**_**10**_ **-PAH**	**PM**_**2.5**_	**PM**_**10**_	**PM**_**10**_ **-PAH**	**PM**_**2.5**_	**PM**_**10**_	**PM**_**10**_ **-PAH**
**Unmethylated CpGs** **in NHBE**	28,627	26,533	28,674	92,202	85,521	99,032	17,746	17,666	19,296	138,575	129,719	144,627
**Unmethylated CpGs** **in NHEK**	17,907	28,937	19,691	40,236	52,578	43,912	6,361	7,565	7,964	64,503	88,747	71,567
**Analyzed total CpGs**	1,784,717			4,454,205			801,773			7,040,695		

## Discussion

The first major finding of this study was that *Alu* and *LINE1* methylation was significantly lower after PM exposures, providing for the first direct experimental evidence that PM exposure induces DNA hypomethylation in NHBE and NHEK. Recent *in vitro* experiments have shown that oxidative DNA damage by PM can interfere with the ability of DNA methyltransferase, resulting in RE hypomethylation [21]. DNA methylation is a common feature of eukaryotic genomes and is a core epigenetic process that influences numerous biological processes, such as gene repression, control of cellular development and differentiation, RE silencing, and maintenance of genome stability [22]. DNA methylation mainly changed at locus-specific and genome-wide levels. A number of methods are available for the analysis of global DNA methylation levels [23]. Recently, WGBS has revolutionized the way of interrogating the methylome to realize genome-wide methylation analysis at a single-base resolution [24]. The current study determined uniquely mappable WGBS data to be the most reproducible and accurate measurement of global DNA methylation levels by comparing with pyrosequencing assays of RE, providing WGBS as the gold standard method in methylomics for its unsurpassed resolution and coverage.

*Alu* is the largest family of short interspersed nuclear elements in the human genome and *LINE1* is a predominant member of LINEs [9]. The former is a non-autonomous, transposable element (TE) to be mobilized in trans by *LINE1*, but the latter is only autonomous TE. Moreover, they constitute the critical regulators of genetic information expression by providing regulatory sequences or introducing alternative start or stop codons into functional genes [25], providing to act as global modifiers of gene expression through changes in their own methylation state. Accordingly, growing evidence has shown that the altered methylation states of TEs might be associated with aging, autoimmune diseases, cardiovascular disease, or cancer development and progression [26], suggesting that these changes are not the simple consequences of the disease, but may often drive the pathogenesis. Interestingly, *Alu* and *LINE1* initiate the spread of CpG island (CGI) methylation and the CGI length is associates with their distribution [27], indicating the potential centers for *de novo* methylation events. Unfortunately, current investigations have focused only on analyzing a single common sequence for *Alu* and *LINE1* through pyrosequencing assays, which is easier to do than previous methods to quantify total genomic 5-methylcytosine [28]. Moreover, recent reports have shown that the methylation of a common sequence is not correlated with global methylation content in normal tissues and that CpG content is a primary determinant of changes over time in DNA methylation at individual CpG sites [29, 30].

The second novel finding of the current study was that the evolutionary age of RE subfamilies determined differential susceptibility of DNA hypomethylation to ambient PM. Sparse data are available on the effects of environmental exposures across different subfamilies of TEs. Based on the peak period of amplification and the level of nucleotide substitutions, *Alu* and *LINE1* are subdivided into each subfamily with different evolutionary ages; young (Alu Y and L1-H), intermediate (Alu S and L1-P), and old (Alu J and L1-M) subfamilies [30, 31]. Because of frequent deamination of methylated cytosines in CpG dinucleotides, older subfamilies remain less rich in CpG sites and show weaker or no transposon activity, whereas young subfamilies are richer in CpGs and still transcriptionally active in the human genome. Moreover, CpG content and DNA methylation levels vary dramatically across subfamilies. Interestingly, Byun et al. [32] have demonstrated that the effect of PM_10_ exposure on DNA methylation depends on the subfamily evolutionary age, with a stronger negative effect on older *LINE1* and younger *Alu*. Recently, older subfamilies of *Alu* and *LINE1* elements (Alu J and L1-M) exhibit great hypomethylation in chronic lymphocytic leukemia [33]. Furthermore, the Alu Y sequence shows remarkable differences in DNA methylation state across colorectal cancer drug resistance [34]. Taken together, these results suggest that the evolutionary age of TE subfamilies might determine differential vulnerability of DNA methylation to environmental exposures.

## Conclusions

The present study showed PM-induced hypomethylation of *Alu* and *LINE1* elements with differential susceptibility of the evolutionary subfamily, suggesting that RE hypomethylation might be a vital mechanism underlying the harmful effects of airborne PM and that monitoring of the methylation status for a specific subset of RE could serve as interface sensors between PM and DNA methylation. Furthermore, these results could provide a better understanding of the effects of PM exposure on RE subfamilies and the role of RE in response to environmental risk factors related to human health and disease. This study is the first to utilize the WGBS platform in the analysis of the subfamily-specific methylation of RE in PM-exposed human skin and lung tissues. However, further work to analyze locus-specific hypomethylation of *Alu* and *LINE1* remains challenging.

## Supplementary information

Additional file 1: **Supplementary Fig. 1. **Graphical overview and representative of *Alu* and *LINE1* data analysis. (A) Workflow chart. (B) Analysis of methylation index in every CG-cytosine using MOABS. (C) Extraction of repetitive elements including *Alu* and *LINE1*. (D) Six repeats (AluJ, AluS, AluY, LiH, L1M, and L1P) extraction using python script. (E) CGs extraction within 6 repeats.

**Additional file 2: Supplementary Table 1.** Overview of WGBS data analysis.

## Data Availability

All data generated or analyzed during this study are included in this published article.

## Abbreviations

DMC: Differentially methylated cytosine
LINE1: Long interspersed nucleotide element 1
NHBE: Normal human bronchial epithelial cells
NHEK: Normal human epidermal keratinocytes
PAH: Polycyclic aromatic hydrocarbons
PM: Particulate matter
RE: Repetitive elements
TE: Transposable element
WGBS: Whole-genome bisulfite sequencing
