# Supplementary figures and images for: Particulate matter-induced hypomethylation of Alu and LINE1 in normal human bronchial epithelial cells and epidermal keratinocytes
Source: Genes Environ. 2022 Feb 16;44:8. doi: 10.1186/s41021-022-00235-4 (PMC8848652; doi:10.1186/s41021-022-00235-4)

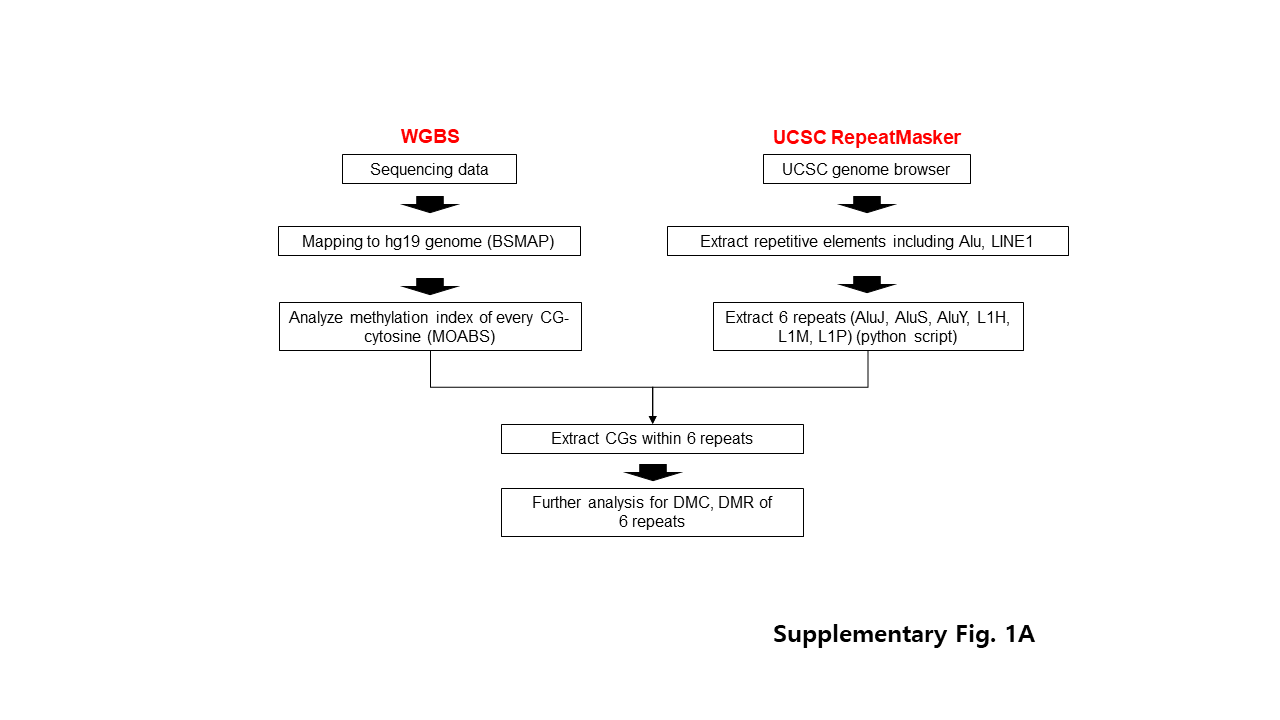

Supplement: Supplementary file 1 — Supplementary Fig. 1. Graphical overview and representative of Alu and LINE1 data analysis. (A) Workflow chart. (B) Analysis of methylation index in every CG-cytosine using MOABS. (C) Extraction of repetitive elements including Alu and LINE1. (D) Six repeats (AluJ, AluS, AluY, LiH, L1M, and L1P) extraction using python script. (E) CGs extraction within 6 repeats. [file 41021_2022_235_MOESM1_ESM.zip › Fig. 1A.TIF]

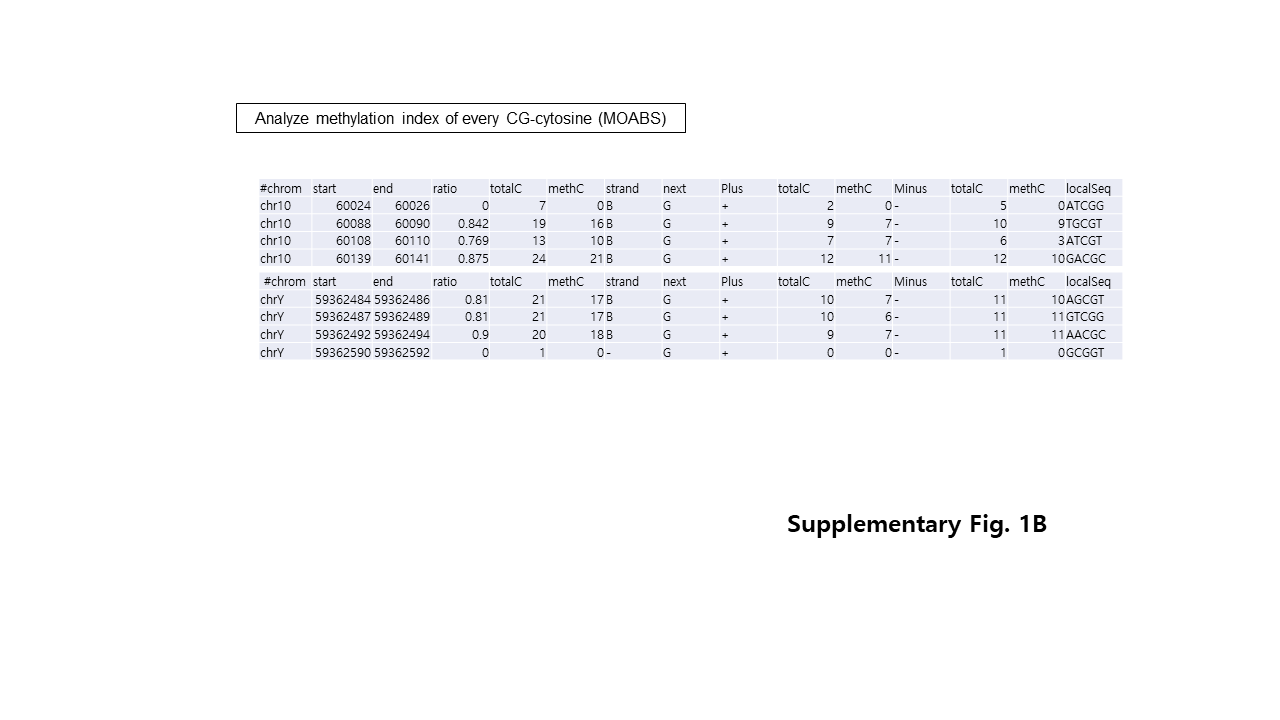

Supplement: Supplementary file 1 — Supplementary Fig. 1. Graphical overview and representative of Alu and LINE1 data analysis. (A) Workflow chart. (B) Analysis of methylation index in every CG-cytosine using MOABS. (C) Extraction of repetitive elements including Alu and LINE1. (D) Six repeats (AluJ, AluS, AluY, LiH, L1M, and L1P) extraction using python script. (E) CGs extraction within 6 repeats. [file 41021_2022_235_MOESM1_ESM.zip › Fig. 1B.TIF]

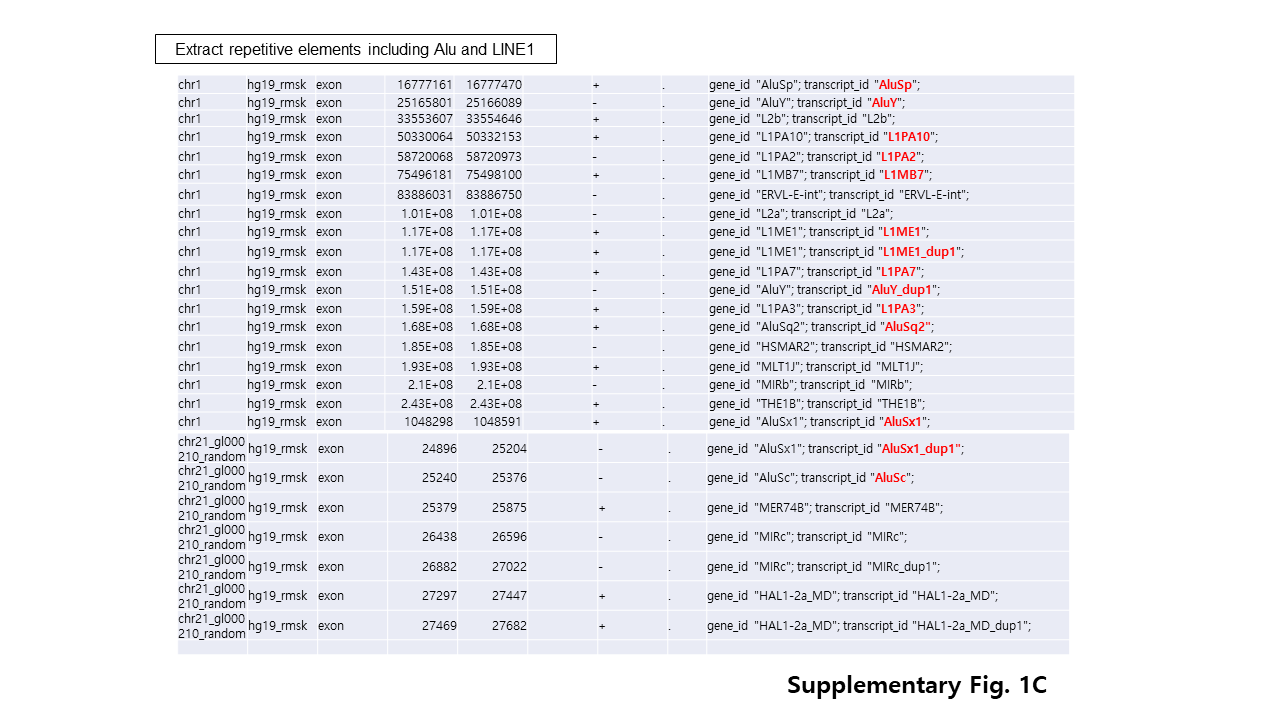

Supplement: Supplementary file 1 — Supplementary Fig. 1. Graphical overview and representative of Alu and LINE1 data analysis. (A) Workflow chart. (B) Analysis of methylation index in every CG-cytosine using MOABS. (C) Extraction of repetitive elements including Alu and LINE1. (D) Six repeats (AluJ, AluS, AluY, LiH, L1M, and L1P) extraction using python script. (E) CGs extraction within 6 repeats. [file 41021_2022_235_MOESM1_ESM.zip › Fig. 1C.TIF]

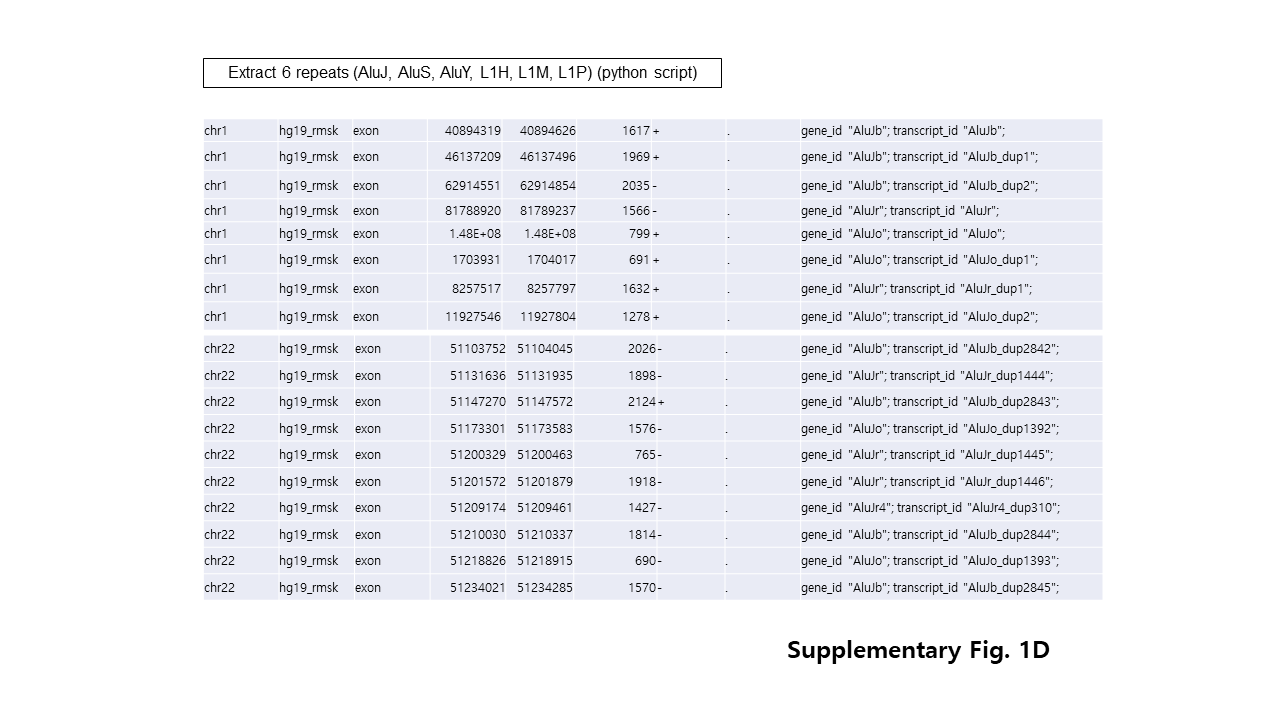

Supplement: Supplementary file 1 — Supplementary Fig. 1. Graphical overview and representative of Alu and LINE1 data analysis. (A) Workflow chart. (B) Analysis of methylation index in every CG-cytosine using MOABS. (C) Extraction of repetitive elements including Alu and LINE1. (D) Six repeats (AluJ, AluS, AluY, LiH, L1M, and L1P) extraction using python script. (E) CGs extraction within 6 repeats. [file 41021_2022_235_MOESM1_ESM.zip › Fig. 1D.TIF]

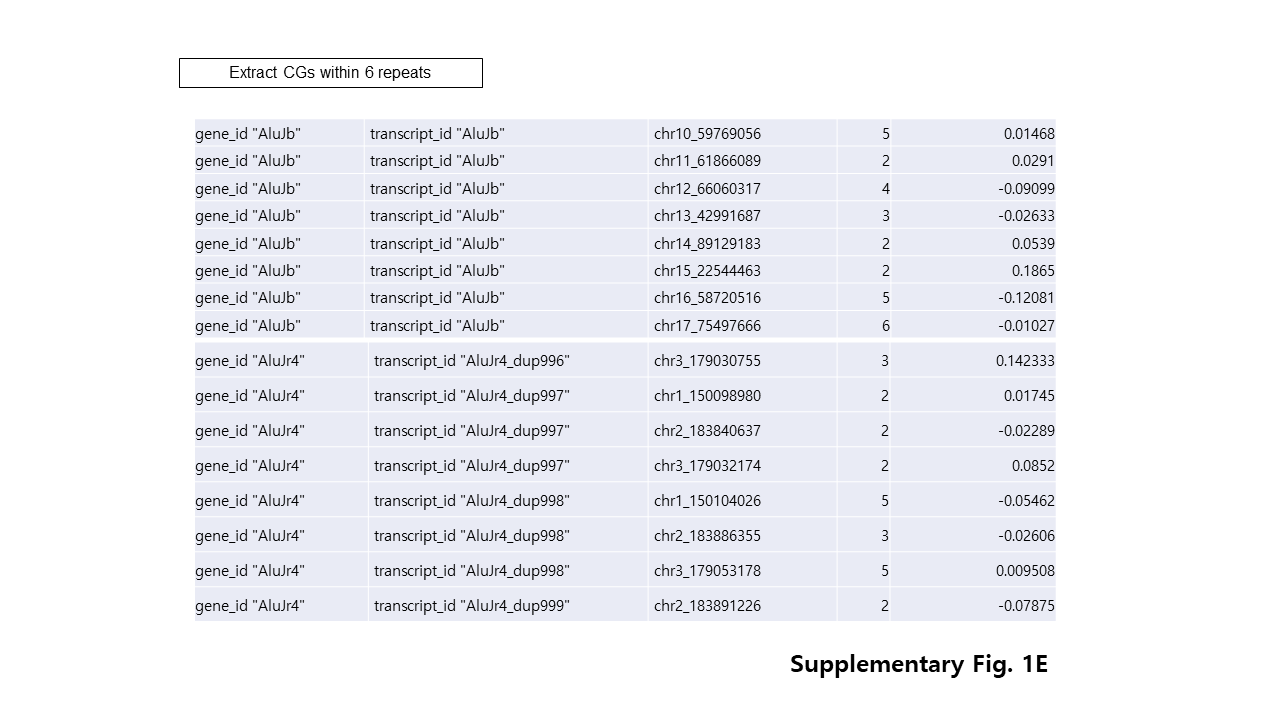

Supplement: Supplementary file 1 — Supplementary Fig. 1. Graphical overview and representative of Alu and LINE1 data analysis. (A) Workflow chart. (B) Analysis of methylation index in every CG-cytosine using MOABS. (C) Extraction of repetitive elements including Alu and LINE1. (D) Six repeats (AluJ, AluS, AluY, LiH, L1M, and L1P) extraction using python script. (E) CGs extraction within 6 repeats. [file 41021_2022_235_MOESM1_ESM.zip › Fig. 1E.TIF]
