# Supplementary material for: Particulate matter-induced hypomethylation of Alu and LINE1 in normal human bronchial epithelial cells and epidermal keratinocytes
Source: Genes Environ. 2022 Feb 16;44:8. doi: 10.1186/s41021-022-00235-4 (PMC8848652; doi:10.1186/s41021-022-00235-4)
Supplement: Supplementary file 2 — Additional file 2: Supplementary Table 1. Overview of WGBS data analysis. [file 41021_2022_235_MOESM2_ESM.docx]

| **Samples** | **Sequencing quantity (nucleotides)** | **Sequencing depth (X)** | **Bisulfite conversion rate (%)** | **Mapped reads (nucleotides)** | **Mapping rate (%)** | **Numbers of**  **CpG site** | **Coverage of CpG site (%)** | **Sequencing depth**  **in CpG site (X)** |
| --- | --- | --- | --- | --- | --- | --- | --- | --- |
| **Human reference genome hg19** | 3,137,161,264 | 1 |  |  |  | 28,217,448 | 100 |  |
| **NHBE** |  |  |  |  |  |  |  |  |
| **CTL** | 110,986,820,800 | 35.38 | 99.05 | 1,019,884,168 | 91.9 | 28,118,734 | 99.65 | 34.96 |
| **PM_2.5_** | 110,455,789,400 | 35.21 | 98.88 | 988,339,814 | 89.5 | 28,125,903 | 99.68 | 32.00 |
| **PM_10_** | 114,067,744,200 | 36.36 | 98.87 | 1,030,902,454 | 90.4 | 28,128,964 | 99.69 | 34.70 |
| **PM_10_-PAH** | 111,230,395,400 | 35.46 | 98.95 | 989,083,665 | 88.9 | 28,089,983 | 99.55 | 34.27 |
| **NHEK** |  |  |  |  |  |  |  |  |
| **CTL** | 111,531,336,200 | 35.55 | 99.05 | 1,035,538,097 | 92.8 | 28,111,777 | 99.63 | 35.27 |
| **PM_2.5_** | 113,221,286,400 | 36.09 | 99.01 | 1,045,215,377 | 92.3 | 28,106,764 | 99.61 | 36.80 |
| **PM_10_** | 110,005,603,000 | 35.07 | 98.97 | 1,009,397,399 | 91.8 | 28,141,182 | 99.63 | 34.41 |
| **PM_10_-PAH** | 110,201,182,000 | 35.13 | 99.02 | 1,008,415,850 | 91.5 | 28,110,155 | 99.62 | 34.98 |

**Supplementary Table 1. Overview of WGBS data analysis.**
